# Cyanidin-3-*O*-Glucoside Mitigates Amyloid-Beta (1–42)-Induced Apoptosis in SH-SY5Y Cells by Regulating Ca^2+^ Homeostasis and Inhibiting Mitochondrial Dysfunction

**DOI:** 10.3390/antiox14040490

**Published:** 2025-04-18

**Authors:** Chao Ma, Yu Nie, Donglei Zhang, Lulu Ran, Su Xu, Xun Ran, Junya Huang, Lingshuai Meng

**Affiliations:** 1 College of Materials Science and Engineering, Guiyang University, Guiyang 550005, China; 2 College of Food Science and Engineering, Guiyang University, Guiyang 550005, China; 3 Guizhou Engineering Research Center for Characteristic Flavor Perception and Quality Control of Drug-Food Homologous Resources, Guiyang University, Guiyang 550005, China

**Keywords:** Cyanidin-3-*O*-glucoside, apoptosis, Ca^2+^ homeostasis, mitochondrial dysfunction

## Abstract

Background: Blueberry anthocyanin such as Cyanidin-3-*O*-glucoside may help prevent Alzheimer’s disease. We aimed to investigate the preventive and therapeutic effects of Cyanidin-3-*O*-glucoside against Aβ_1–42_-induced apoptosis of SH-SY5Y cells as well as the underlying mechanisms. Methods: Cell viability and intracellular and mitochondrial reactive oxygen species were detected by MTT, a reactive oxygen species detection kit, and a MitoSOX red mitochondrial superoxide indicator. The mitochondrial membrane potential, intracellular calcium ion content, and adenotriphophate (ATP) were identified via a mitochondrial membrane potential detection kit, calcium ion detection kit, and ATP detection kit, and apoptosis was detected via flow cytometry. Transcription of apoptosis-related genes was detected using real-time fluorescence quantitative polymerase chain reaction, and expression of apoptosis-related proteins was identified using Western blot. Results: We found that Cyanidin-3-*O*-glucoside could downregulate the expression of cytochrome c, caspase 9, caspase 3, and other genes and proteins, which consequently reduced the rate of apoptosis. Additionally, it could upregulate Bcl-2 gene and protein expression, downregulate Bax gene and protein expression, regulate mitochondrial membrane permeability and calcium-release channels, reduce calcium influx into mitochondria, maintain intracellular calcium ion levels, reduce intracellular levels of reactive oxygen species and increase ATP levels, maintain the mitochondrial membrane potential at a normal level, maintain normal mitochondrial functioning, and prevent apoptosis. Discussion: Taken together, Cyanidin-3-*O*-glucoside showed dose-dependent preventive and therapeutic effects against Aβ_1–42_-induced apoptosis of SH-SY5Y cells. Conclusions: Cyanidin 3-*O*-glucoside showed a better preventive effect than therapeutic effect against Aβ_1–42_-induced apoptosis in SH-SY5Y cells.

## 1. Introduction

Economic development and societal progress have improved living standards. Consistent with the aging population, Alzheimer’s disease (AD) has become the most prevalent age-related disease among individuals aged ≥ 65 years. In the United States, 5.8 million people have AD, and this number is projected to reach 13.8 million by 2050 [1,2]. In 2017, there were 121,404 deaths related to AD; moreover, mortality due to stroke, heart disease, and prostate cancer increased by 145% between 2000 and 2017. In 2018, >16 million family members and other unpaid caregivers provided approximately 18.5 billion hours of care to patients with AD or other dementias. The cost of this care, which was valued at approximately USD 234 billion, increases the financial burden as well as the risk of adverse physical and mental health outcomes of home caregivers. In 2019, the total cost of medical, long-term care, and hospice services for patients aged 65 years with dementia was estimated at USD 290 billion [3,4]. Accordingly, it is more important to effectively prevent AD than to treat it.

According to the Alzheimer’s Association, AD diagnosis is challenging; moreover, diet is associated with AD [5]. B vitamins and antioxidants exert beneficial effects on brain synapses [6]. Blueberries are well-known for their nutritional value and are rich in natural water-soluble pigments. Worldwide, they are among the top five healthiest fruits since they are rich in phenolic substances, such as anthocyanins. Blueberry fruit extract is a major component of numerous pharmaceutical and food supplement products. Additionally, blueberry anthocyanins have been clinically used for ophthalmological, blood vessel, and connective tissue disorders, as well as diabetes [7]. Specifically, blueberry anthocyanins have shown preventive effects against common diseases, including liver cancer, breast cancer, diabetes, and eye diseases [8,9,10,11]. Anthocyanin accumulation was dependent on the interaction between the cultivar and area [12]. Currently, blueberries are widely grown in China, including *Vaccinium uliginosum* in northeast China, *V. bracteatum* in southwest China, and *V. nigrum* in Jiangnan. Furthermore, Guizhou Province in southern China is the most important region in terms of blueberry growing. Compared with winters in other places worldwide on the same latitude, those in the Yangtze River basin and southern China are not severe. Guizhou Province has abundant rainfall, sufficient light and heat, four distinct seasons, and a large area of acid soil [13], which is similar to conditions in blueberry-growing areas in the Middle East and southern United States [14]. The anthocyanin profiles of fruits of four blueberry cultivars (*V. bracteatum*) from Guizhou (Gardenblue, Legacy, Misty, and Brightwell) were investigated in the previous study, which showed that blueberries from Guizhou contain abundant anthocyanin resources and deserve further in-depth research [15].

Blueberry anthocyanin extracts have shown potential effects against AD [16]; however, there are currently few reports on the preventive and therapeutic effects of Cyanidin-3-*O*-glucoside on Alzheimer’s disease, and related research showed that Cyanidin-3-*O*-glucoside attenuates amyloid-beta (1–40)-induced oxidative stress and apoptosis in SH-SY5Y cells through a Nrf2 mechanism [17], but the effect of Cyanidin-3-*O*-glucoside on AD by regulating Ca^2+^ homeostasis and mitochondrial dysfunction has not been reported. Therefore, we aimed to investigate the effect of Cyanidin-3-*O*-glucoside on amyloid-beta (1–42)-induced apoptosis in SH-SY5Y cells by regulating Ca^2+^ homeostasis and inhibiting mitochondrial dysfunction and then evaluate the preventive and treatment effects of Cyanidin-3-*O*-glucoside on AD.

## 2. Materials and Methods

### 2.1. Chemicals and Reagents

**Figure 1:**
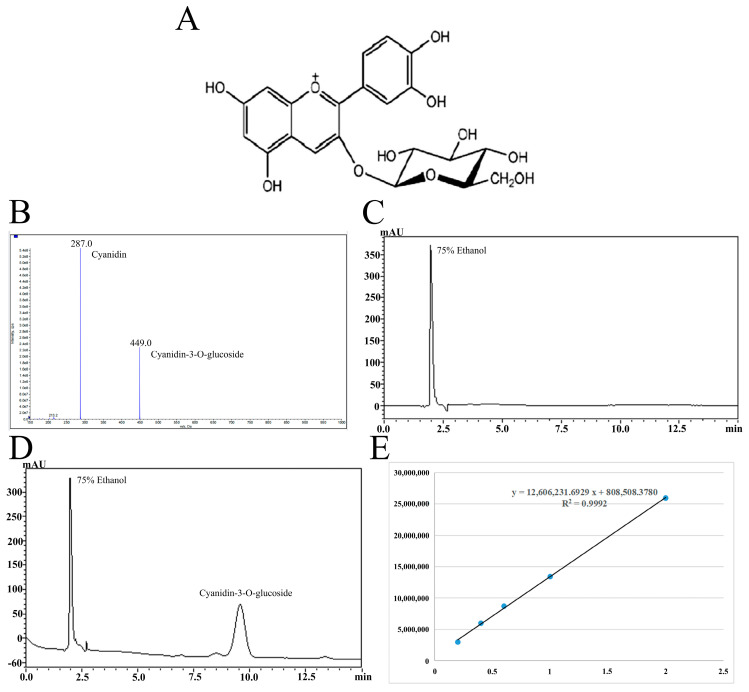
The chemical structure of Cyanidin-3-*O*-glucoside (**A**). Mass spectrometry (**B**) and liquid chromatography (**C**,**D**) of Cyanidin-3-*O*-glucoside purified by high-performance liquid preparation chromatography and standard curve of Cyanidin-3-*O*-glucoside (**E**).

Cyanidin-3-*O*-glucoside (93.73% purity) was isolated from blueberries by acidic ethanol combined with ultrasound and purified by AB-8 macroporous resin combined with preparative high-performance liquid chromatography. The specific extraction and purification processes refer to the research of Ma et al. [18], and the chemical structure and the qualitative and quantitative analysis results of Cyanidin-3-*O*-glucoside are shown in Figure 1. Table 1 shows the drugs and reagents used in the experiment while Table 2 shows the instruments and equipment used.

### 2.2. SH-SY5Y Cell Culture and Cell Grouping

The SH-SY5Y neuroblastoma cell line was obtained from Wanlei Biological Technology Co., Ltd. (Shenyang, China), and the SH-SY5Y human neuroblastoma cells were cultured on Minimum Essential Medium (MEM)/F-12 complete medium (43.5% MEM, 43.5% F-12, 10% fetal bovine serum, 1% dual antibody (Penicillin-Streptomycin), 1% Sodium Pyruvate, 1% GLUTA-max, and 1% non-essential amino acids). The cells were cultured in an incubator with 5% CO_2_ at 37 °C. Cells at the logarithmic growth stage were selected for subsequent experiments. A reference was made for experimental grouping [19], with specific processes being performed according to the group as follows:

1. A. Normal control group;
2. B. Solvent control group: 0.3% Dimethylsulfoxide (DMSO) was used to dissolve Aβ_1–42_ and Cyanidin-3-*O*-glucoside;
3. C. Cell model group: cells were incubated with 1 µM Aβ_1–42_ for 24 h;
4. D. Low-dose drug protection group: cells were pre-treated with 20 µg/mL Cyanidin-3-*O*-glucoside for 24 h, followed by incubation with 1 µM Aβ_1–42_ for 24 h, and Cyanidin-3-*O*-glucoside is left during Aβ_1–42_ incubation;
5. E. Medium drug protection group: cells were pre-treated with 40 µg/mL Cyanidin-3-*O*-glucoside for 24 h, followed by incubation with 1 µM Aβ_1–42_ for 24 h, and Cyanidin-3-*O*-glucoside is left during Aβ_1–42_ incubation;
6. F. High-dose drug protection group: cells were pre-treated with 60 µg/mL Cyanidin-3-*O*-glucoside for 24 h, followed by incubation with 1 µM Aβ_1–42_ for 24 h, and Cyanidin-3-*O*-glucoside is left during Aβ_1–42_ incubation;
7. G. Drug treatment group: cells were incubated with 40 µg/mL Cyanidin-3-*O*-glucoside and 1 µM Aβ_1–42_ for 24 h, and Cyanidin-3-*O*-glucoside is left during Aβ_1–42_ incubation.

### 2.3. Cell Viability Was Detected by MTT Assay

According to the experimental groups, the cells were seeded in 96-well plates at a density of 5 × 10^3^ cells per well and cultured in a 37 °C, 5% CO_2_ incubator. Cell viability was examined using the MTT assay. Here, each cell group was photographed at a specific time and the culture medium was discarded. Next, culture medium containing 20 µL MTT staining solution was added to each well and incubated at 37 °C for 4 h in a 5% CO_2_ incubator. After 4 h, the supernatant was carefully removed and 150 µL DMSO was used to dissolve the purple crystals formed by the cells. The optical density value at 570 nm was measured on a microplate analyzer after standing for 10 min in darkness.

### 2.4. Detection of Intracellular Reactive Oxygen Species (ROS) by Reactive Oxygen Species Detection Kit

According to the experimental groups, the cells were seeded in 96-well plates at a density of 5 × 10^3^ cells per well and cultured in a 37 °C, 5% CO_2_ incubator. Each cell group was collected and washed twice using phosphate-buffered saline (PBS). The dichloro-dihydro-fluorescein diacetate (DCFH-DA) diluent of 1 mL (diluted with medium according to 1:1000) was added to the mix well and incubated for 20 min at 37 °C with alternate inversions at 5 min intervals. The cells were washed thrice with PBS, followed by complete removal of the DCF—DA. Flow cytometry was performed after the cells were resuspended with 500 µL PBS.

### 2.5. Detection of Mitochondrial ROS by MitoSOX Red Mitochondrial Superoxide Indicator

Each group of cells was collected and washed twice with PBS. Then, 50 μg of the probe was dissolved in 13 μL DMSO to form a 5 mM stock solution. Then, a 5 μM working solution (diluted 1:1000 with PBS) was added to each group of cells and incubated at 37 °C for 10 min. The cells were washed, resuspended in 500 μL PBS, and analyzed by flow cytometry.

### 2.6. Detection of Mitochondrial Membrane Potential Using Mitochondrial Membrane Potential Detection Kit

According to the experimental groups, the cells were seeded in 96-well plates at a density of 5 × 10^3^ cells per well and cultured in a 37 °C, 5% CO_2_ incubator. Each cell group was collected and washed twice using PBS. Next, 0.5 mL JC-1 staining working solution was added and mixed well. The cells were incubated at 37 °C for 20 min. During incubation, we prepared an appropriate amount of JC-1 dyeing buffer (1×) by diluting 2 mL JC-1 dyeing buffer (5×) with 8 mL distilled water. The cells were collected by centrifugation at 600× *g* for 3 min, the supernatant was discarded by centrifugation, and the cells were washed twice with JC-1 staining buffer (1×). Next, the cells were resuspended in JC-1 staining buffer (1×), followed by detection of the mitochondrial membrane potential.

### 2.7. Detection of Cellular ATP Levels by ATP Detection Kit

According to the experimental groups, the cells were seeded in 96-well plates at a density of 5 × 10^3^ cells per well and cultured in a 37 °C, 5% CO_2_ incubator. The cells of each group were collected and lysed using 200 μL ATP lysis solution. The cells were centrifuged for 5 min at 12,000× *g* at 4 °C. The supernatant was used for follow-up detection. Regarding the preparation of the standard curve, we performed concentration gradient dilution of the ATP standard sample using ATP detection lysate. The diluted concentrations were as follows: 0.01, 0.03, 0.1, 0.3, 1, 3, and 10 μmol/L. To prepare the ATP detection working solution, the ATP detection reagent diluent was used to dilute the ATP detection reagent at a ratio of 1:9. Specifically, we added 100 μL of ATP detection working solution per well and left it standing at room temperature for 5 min to reduce the background ATP. Next, 20% ATP detection working fluid was added and mixed well, followed by immediate testing. ATP levels were calculated based on the standard curve and expressed as ATP/sample protein concentration (nmol/mg protein).

### 2.8. Detection of Apoptosis Using Flow Cytometry

According to the experimental groups, the cells were seeded in 96-well plates at a density of 5 × 10^3^ cells per each well and cultured in a 37 °C, 5% CO_2_ incubator with saturated humidity. Subsequently, cells of each group were collected after centrifugation at 300× *g* for 5 min. The supernatant was carefully removed, followed by washing the cells twice with PBS and counting. Next, 10^5^ cells were collected and 500 μL binding buffer was added to the resuspension, followed by the addition of 5 μL Annexin V-FITC 1 × Binding Buffer. We added 10 μL propidium iodide 1 × Binding Buffer, followed by incubation at room temperature in complete darkness for 15 min.

### 2.9. Cellular Calcium Levels

Intracellular calcium levels were measured using a calcium ion colorimetric kit (C004, Nanjing Jiancheng Co., Ltd., Nanjing, China). First, 1 × 10^6^ cells were collected at each concentration, diluted with 100 μL PBS containing 1% protease inhibitor, centrifuged for 10 min at 12,000 rpm, and the supernatant was collected. The 500 mM standard solution was diluted to 5 mM; subsequently, 0, 2, 4, 6, 8, and 10 μL of the diluted standard was added to 96-well plates, with water being added to achieve a final volume of 50 μL per well. We added 25 μL to each sample well, with water being added to reach a final volume of 50 μL. Next, we added 90 μL of chromogenic reagent to each well and mixed thoroughly. Subsequently, 60 μL of calcium solution was added to each well and was incubated at room temperature in the dark for 5–10 min. Detection was performed at 575 nm using a microplate reader (SpectraMax iD5, Beijing Yuechanghang Technology Co., Ltd., Beijing, China). The standard curve was drawn based on the concentration of the standard; subsequently, the calcium concentration of each well was calculated based on the standard curve. The sample pore concentration (μg/μL) was calculated as follows: concentration of each sample well/sample volume added to each well. Further, the sample concentration (μg/μL) was calculated as follows: sample pore concentration μg/μL × 40.

### 2.10. Western Blot

The protein concentration was determined using the BCA protein determination kit. Proteins were separated through Sodium Dodecyl Sulphate-Polyacrylamide Gel Electrophoresis; subsequently, the membrane was transferred. Polyvinylidene fluoride membrane was used as solid support. Next, the membrane film was soaked from bottom to top with PBS/Tween (PBST) and moved to a plate containing a sealing solution, which was placed on a shaking table at room temperature for 1.5 h. The primary antibody (cytochrome c, caspase 9, cleaned caspase 3, and Bcl-2, Bax) dissolved in PBST was diluted to an appropriate concentration (in a 1.5 mL centrifuge tube). An appropriately sized piece of fresh-keeping film was torn off and laid on the test table. The four corners were soaked with water to keep the film flat, followed by the addition of the antibody solution. The membrane was removed from the sealing solution, and the residual solution was absorbed using filter paper. The membrane protein was placed at the antibody liquid level; moreover, the four membrane corners were lifted to remove the residual bubbles. After incubating at 4 °C overnight, the membrane was washed thrice (10 min sessions) with PBST at room temperature on a shaking bed. The secondary antibody diluent (1:2000) was prepared as aforementioned and added to the membrane. After incubation at room temperature for 1.5 h, the membrane was washed thrice (10-min sessions) with PBST at room temperature on a shaking bed. Two reagents, A and B, were mixed in equal volumes. The film was laid on the distribution board and the mixed solution was poured onto the film until it was completely covered. Subsequently, the film was placed into the ECL luminescent instrument to emit light, and the picture was saved. Finally, the net optical density of the target band was analyzed using a gel image processing system.

### 2.11. Total RNA Extraction

TRI pure lysate (1 mL) was added to the sample, mixed well, and incubated at room temperature for 5 min. Next, 200 µL of the solution was mixed with chloroform and allowed to stand at room temperature for 3 min. The solution was centrifuged at 10,000× *g* (4 °C) for 10 min and divided into the organic phase, intermediate layer, and colorless aqueous phase. The aqueous phase was transferred into a new centrifuge tube. Similar volumes of isopropanol and water were mixed and placed at −20 °C overnight. After 15 h, the mixture solution was centrifuged at 10,000× *g* (4 °C) for 10 min and the supernatant was discarded. Next, 1 mL of 75% ethanol was added, and the mixture was centrifuged at 3400× *g* (4 °C) for 3 min, followed by the removal of the supernatant. Subsequently, the solution was allowed to stand at room temperature for 5–6 min, and the residual ethanol was allowed to volatilize. Next, 30 µL of Rnase-free ddH_2_O was added, and the solution was kept at room temperature for 2 min, mixed extensively, and allowed to stand until the precipitate completely dissolved to yield the total RNA of the sample. The RNA concentration in each sample was determined using a UV spectrophotometer (NANO 2000, Thermo, Beijing, China).

### 2.12. Reverse Transcription

The obtained RNA samples were reverse transcribed to obtain the corresponding cDNA using a Takara kit (AMV, Thermo, Beijing, China). The total reverse transcription system used was 10 µL. The rapid reverse transcription reaction steps were as follows: reverse transcription reaction at 42 °C for 15 min, inactivation at 80 °C for 15 s, and maintenance at 4 °C. The cDNA obtained from reverse transcription at −20 °C were used for subsequent analysis.

### 2.13. Real-Time Fluorescence Quantitative Analysis

The cDNA obtained by reverse transcription was used as a template and was amplified using fluorescence quantitative PCR (Exicycler 96, BIONEER, Beijing, China). A 20 µL reaction system was utilized. We used 1 µL and 0.5 µL of the cDNA template for the upstream and downstream primers, respectively (10 µM). Moreover, 10 µL SYBR GREEN master mix and ddH_2_O were added until a total concentration of 20 µL was reached. The reaction steps were as follows: 94 °C for 5 min; 40 cycles of 94 °C for 10 s, 60 °C for 20 s, and 72 °C for 30 s; and 4 °C for 5 min. Finally, we performed quantitative fluorescence analysis using the ExicyclerTM 96 fluorescence quantitative instrument (ExicyclerTM 96, BIONEER, Beijing, China).

### 2.14. Statistical Analyses

The mean value and standard deviation were calculated using Microsoft Excel. Statistical analyses were performed using SPSS software (version 17.0). Between-group comparisons were performed using a one-way analysis of variance. *p* < 0.05 was considered significant, and *p* < 0.01 was extremely significant. Drawing was performed using Origin 7.5 software.

## 3. Results

### 3.1. Cell Viability

**Figure 2:**
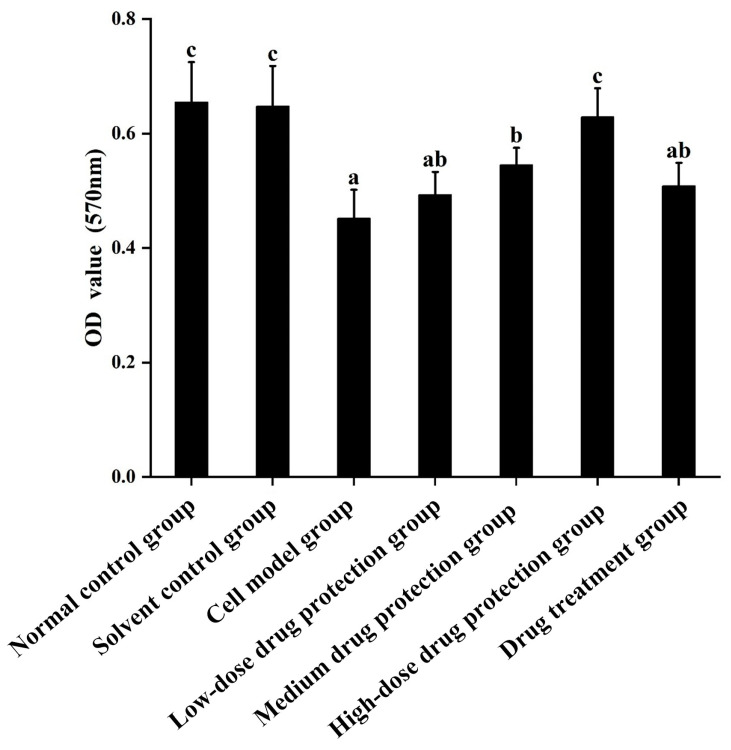
The protective and therapeutic effects of Cyanidin-3-*O*-glucoside on SH-SY5Y cell injury induced by Aβ_1–42_. Solvent control group: 0.3% Dimethylsulfoxide (DMSO); cell model group: cells were incubated with 1 µM Aβ_1–42_ for 24 h; low/medium/high-dose drug protection group: cells were pre-treated with 20/40/60 µg/mL Cyanidin-3-*O*-glucoside for 24 h, followed by incubation with 1 µM Aβ_1–42_ for 24 h, and Cyanidin-3-*O*-glucoside was left during Aβ_1–42_ incubation; drug treatment group: cells were incubated with 40 µg/mL Cyanidin-3-*O*-glucoside and 1 µM Aβ_1–42_ for 24 h, and Cyanidin-3-*O*-glucoside was left during Aβ_1–42_ incubation. Different lowercase letters indicate significant differences (*p* < 0.05).

Cell viability was measured using the MTT method. The results are shown in Figure 2. Compared with the control group, the Aβ_1–42_ treatment (model) group showed significantly decreased cell viability (*p* < 0.05), which decreased by 30.99%. Moreover, 20 μg/mL Cyanidin-3-O-glucoside did not significantly increase cell viability (*p* > 0.05), indicating no significant protective effect. However, 40 μg/mL Cyanidin-3-O-glucoside significantly increased cell viability (*p* < 0.05), with 60 μg/mL Cyanidin-3-O-glucoside showing the best protective effect. Taken together, Cyanidin-3-O-glucoside showed a dose-dependent preventive effect against the Aβ_1–42_-induced decline in cell viability.

### 3.2. Determination of ROS

#### 3.2.1. Intracellular ROS

**Figure 3:**
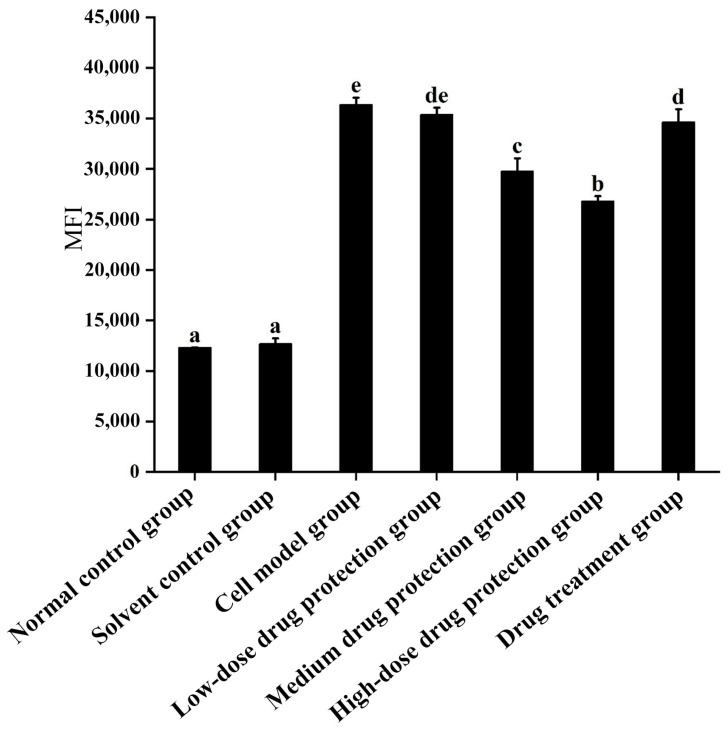
Detection of intracellular levels of reactive oxygen species. Solvent control group: 0.3% Dimethylsulfoxide (DMSO); cell model group: cells were incubated with 1 µM Aβ_1–42_ for 24 h; low/medium/high-dose drug protection group: cells were pre-treated with 20/40/60 µg/mL Cyanidin-3-*O*-glucoside for 24 h, followed by incubation with 1 µM Aβ_1–42_ for 24 h, and Cyanidin-3-*O*-glucoside was left during Aβ_1–42_ incubation; drug treatment group: cells were incubated with 40 µg/mL Cyanidin-3-*O*-glucoside and 1 µM Aβ_1–42_ for 24 h, and Cyanidin-3-*O*-glucoside was left during Aβ_1–42_ incubation. Different lowercase letters indicate significant differences (*p* < 0.05).

As shown in Figure 3, compared with the control group, the model group showed significantly increased oxygen free radicals. Additionally, there was no difference in the ROS between the model group and low dose drug protection group (*p* > 0.05). Cyanidin-3-*O*-glucoside showed a dose-dependent decreasing effect on the levels of ROS in cells, with a dose of 60 μg/mL showing the lowest levels of ROS. Additionally, compared with the model group, the treatment group showed significantly lower oxygen free radicals compared with the model group and significantly higher levels compared to the middle dose drug protection group (both, *p* < 0.05). Taken together, Cyanidin-3-*O*-glucoside can mitigate the oxidative stress induced by Aβ_1–42_ in SH-SY5Y cells. At the same dose, Cyanidin-3-*O*-glucoside showed a better preventive and protective effect than its therapeutic effect.

#### 3.2.2. Mitochondrial ROS

**Figure 4:**
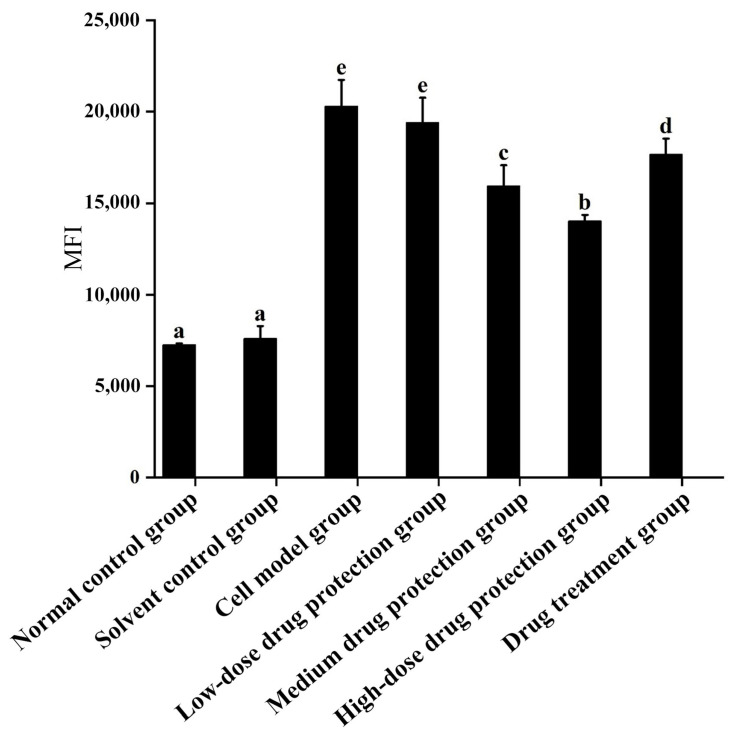
Detection of mitochondrial levels of reactive oxygen species. Solvent control group: 0.3% Dimethylsulfoxide (DMSO); cell model group: cells were incubated with 1 µM Aβ_1–42_ for 24 h; low/medium/high-dose drug protection group: cells were pre-treated with 20/40/60 µg/mL Cyanidin-3-*O*-glucoside for 24 h, followed by incubation with 1 µM Aβ_1–42_ for 24 h, and Cyanidin-3-*O*-glucoside was left during Aβ_1–42_ incubation; drug treatment group: cells were incubated with 40 µg/mL Cyanidin-3-*O*-glucoside and 1 µM Aβ_1–42_ for 24 h, and Cyanidin-3-*O*-glucoside was left during Aβ_1–42_ incubation. Different lowercase letters indicate significant differences (*p* < 0.05).

As shown in Figure 4, compared with the control group, the model group showed significantly increased oxygen free radicals in the mitochondria of SH-SY5Y cells. Additionally, there was no difference in the oxygen free radicals between the model group and low-dose drug protection group (*p* > 0.05). Cyanidin-3-*O*-glucoside showed a dose-dependent decreasing effect on the levels of oxygen free radicals in cells, with a dose of 60 μg/mL showing the lowest level of oxygen free radicals. The treatment group showed significantly lower levels of oxygen free radicals than the model group and significantly higher levels than in medium-dose drug protection group (both, *p* < 0.05). Taken together, Cyanidin-3-*O*-glucoside can mitigate the oxidative stress induced by Aβ_1–42_ in the mitochondria of SH-SY5Y cells. At the same dose, Cyanidin-3-*O*-glucoside showed a better preventive and protective effect than its therapeutic effect.

### 3.3. Mitochondrial Membrane Potential

JC-1 is a cationic lipid fluorescent dye that can be used as an indicator for detecting mitochondrial transmembrane potential. In normal cells, when the membrane potential is normal, JC-1 enters the mitochondria through the polarity of the mitochondrial membrane and forms a polymer that emits red fluorescence due to increased concentration. In apoptotic cells, the mitochondrial transmembrane potential is depolarized, and JC-1 is released from the mitochondria, decreasing in concentration and reversing to a monomeric form that emits green fluorescence. Therefore, changes in mitochondrial membrane potential can be detected by detecting green and red fluorescence.

**Figure 5:**
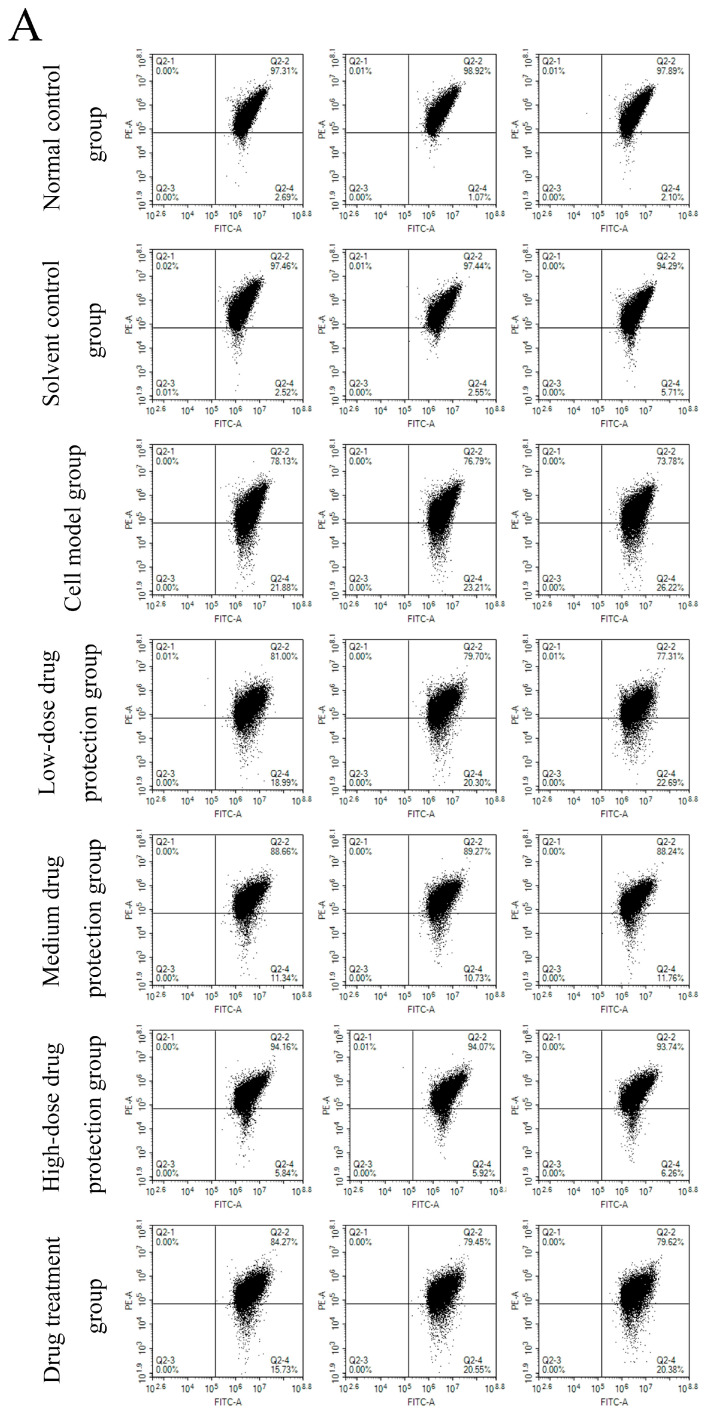

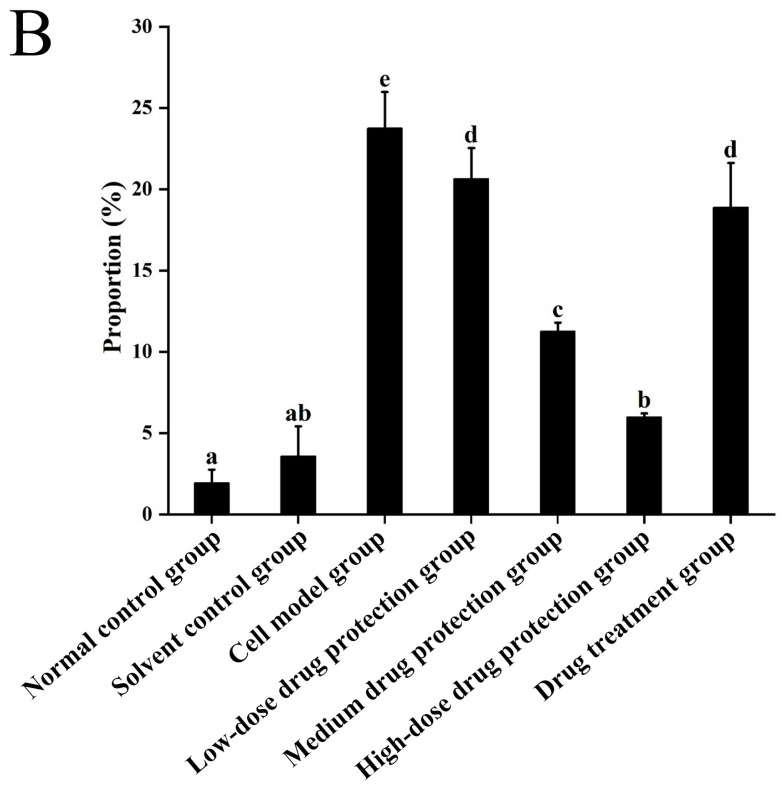
Determination of mitochondrial membrane potential ((**A**): the distribution of red fluorescence and green fluorescence in each quadrant (**B**): the proportion of green fluorescence). Solvent control group: 0.3% Dimethylsulfoxide (DMSO); cell model group: cells were incubated with 1 µM Aβ_1–42_ for 24 h; low/medium/high-dose drug protection group: cells were pre-treated with 20/40/60 µg/mL Cyanidin-3-*O*-glucoside for 24 h, followed by incubation with 1 µM Aβ_1–42_ for 24 h, and Cyanidin-3-*O*-glucoside was left during Aβ_1–42_ incubation; drug treatment group: cells were incubated with 40 µg/mL Cyanidin-3-*O*-glucoside and 1 µM Aβ_1–42_ for 24 h, and Cyanidin-3-*O*-glucoside was left during Aβ_1–42_ incubation. Different lowercase letters indicate significant differences (*p* < 0.05).

The results are shown in Figure 5A, showing the changes in red fluorescence and green fluorescence in the different groups. Figure 5B shows the changes in green fluorescence across the different groups. The proportions of green fluorescence in the normal and solvent control groups were 1.95% and 3.95%, respectively. The model group had a significantly higher proportion of green fluorescence (23.77%) than the control group (*p* < 0.05), indicating that Aβ_1–42_ can induce SH-SY5Y cells to reduce mitochondrial membrane potential. In the drug protection groups, the dose of Cyanidin-3-*O*-glucoside was negatively correlated with the proportion of green fluorescence. The high-dose drug protection group had the lowest proportions of green fluorescence, but it was higher than the solvent control group (*p* < 0.05). Additionally, the treatment group showed a significantly lower proportion of green fluorescence (18.89%) than the model group and a higher proportion than in the medium-dose drug protection group (both *p* < 0.05). This shows that Cyanidin-3-*O*-glucoside can protect SH-SY5Y cells from the Aβ_1–42_-induced decrease in mitochondrial membrane potential in SH-SY5Y cells and showed a significantly better protective effect than its therapeutic effect (*p* < 0.05).

### 3.4. Intracellular Calcium Levels

**Figure 6:**
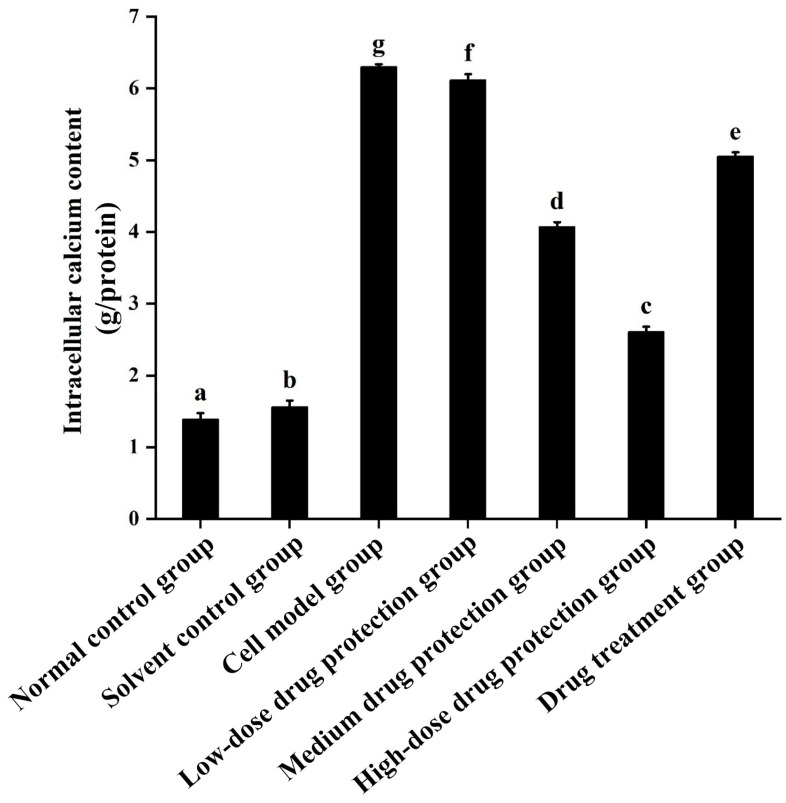
Determination of intracellular Ca^2+^ levels. Solvent control group: 0.3% Dimethylsulfoxide (DMSO); cell model group: cells were incubated with 1 µM Aβ_1–42_ for 24 h; low/medium/high-dose drug protection group: cells were pre-treated with 20/40/60 µg/mL Cyanidin-3-*O*-glucoside for 24 h, followed by incubation with 1 µM Aβ_1–42_ for 24 h, and Cyanidin-3-*O*-glucoside was left during Aβ_1–42_ incubation; drug treatment group: cells were incubated with 40 µg/mL Cyanidin-3-*O*-glucoside and 1 µM Aβ_1–42_ for 24 h, and Cyanidin-3-*O*-glucoside was left during Aβ_1–42_ incubation. Different lowercase letters indicate significant differences (*p* < 0.05).

As shown in Figure 6, compared with the control group, the model group showed a significantly higher concentration of calcium ions (6.304 mmol/g prot, *p* < 0.05). Compared with the model group, the drug protection groups showed a dose-dependent decrease in calcium levels (*p* < 0.05), with the calcium levels in the high-dose drug protection group being 2.608 mmol/g prot. The treatment group showed significantly lower intracellular calcium levels than the model group and higher intracellular calcium levels (5.056 mmol/g prot, *p* < 0.05) than the medium-dose drug protection group. This indicates that Cyanidin-3-*O*-glucoside can mitigate the Aβ_1–42_-induced increase in calcium ions in SH-SY5Y cells. Further, it showed a significantly better preventive effect than its therapeutic effect.

### 3.5. ATP

**Figure 7:**
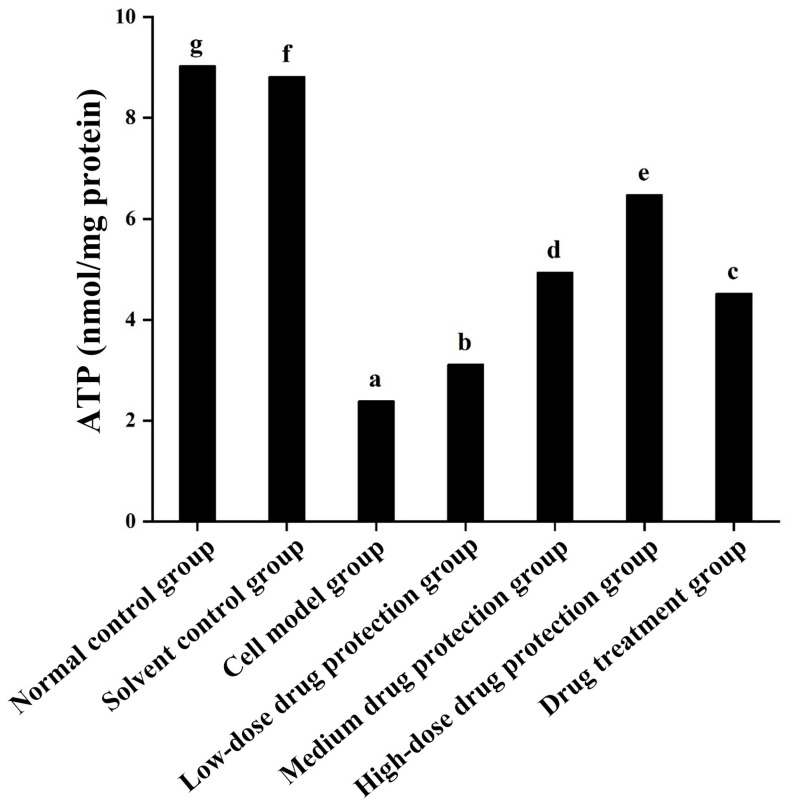
Detection of ATP. Solvent control group: 0.3% Dimethylsulfoxide (DMSO); cell model group: cells were incubated with 1 µM Aβ_1–42_ for 24 h; low/medium/high-dose drug protection group: cells were pre-treated with 20/40/60 µg/mL Cyanidin-3-*O*-glucoside for 24 h, followed by incubation with 1 µM Aβ_1–42_ for 24 h, and Cyanidin-3-*O*-glucoside was left during Aβ_1–42_ incubation; drug treatment group: cells were incubated with 40 µg/mL Cyanidin-3-*O*-glucoside and 1 µM Aβ_1–42_ for 24 h, and Cyanidin-3-*O*-glucoside was left during Aβ_1–42_ incubation. Different lowercase letters indicate significant differences (*p* < 0.05).

As shown in Figure 7, compared with the normal and solvent control groups (9.030 and 8.823 μmol/L, respectively), the model group (2.391 μmol/L) showed significantly lower ATP levels. Compared with the model group, the drug protection groups showed a dose-dependent increase in ATP levels (*p* < 0.05), with the ATP levels in the drug protection group being 6.481 μmol/L. The treatment group showed significantly higher intracellular ATP levels than the model group and lower intracellular ATP levels (4.525 μmol/L, *p* < 0.05) than the drug protection group. This suggests that Cyanidin-3-*O*-glucoside can mitigate the Aβ_1–42_ induced decrease in ATP levels in SH-SY5Y cells. Further, it showed a significantly better preventive effect than its therapeutic effect.

### 3.6. Cell Apoptosis

**Figure 8:**
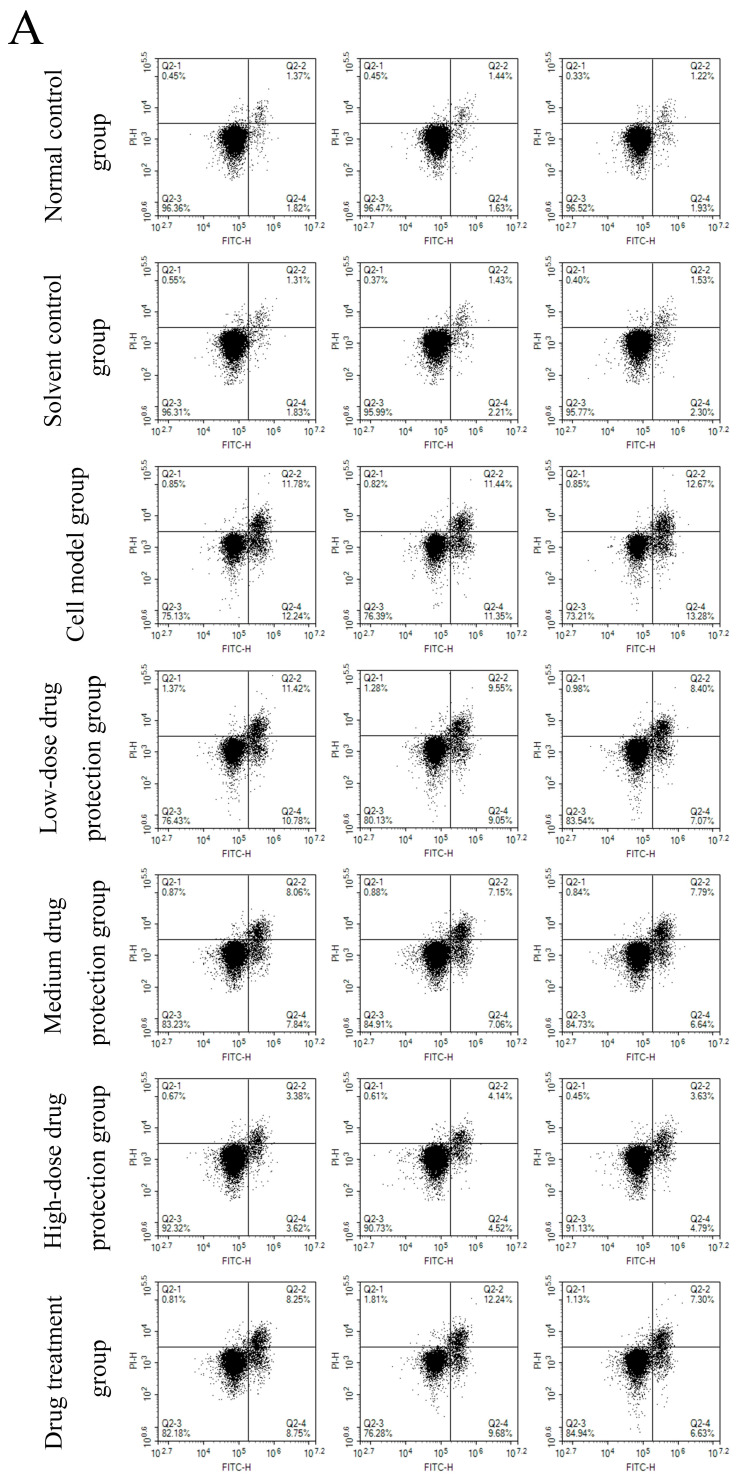

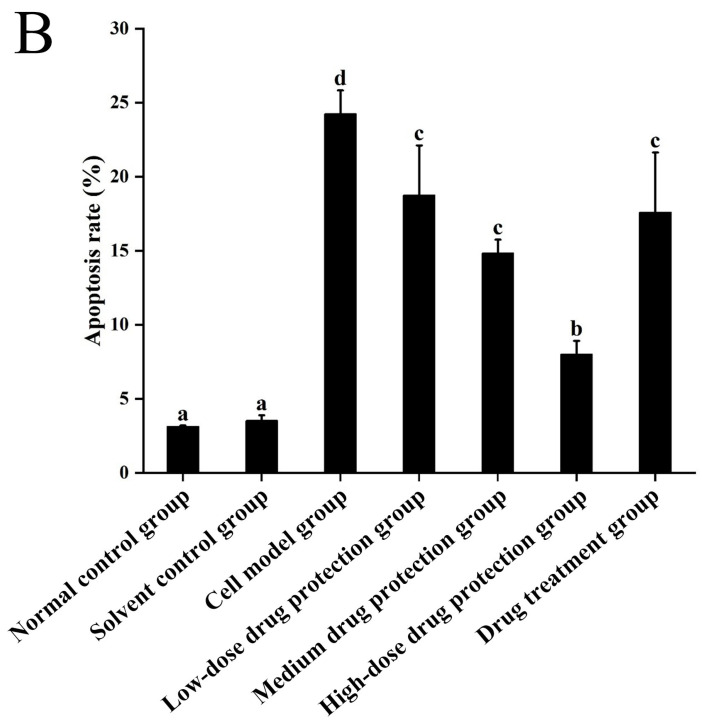
Apoptosis detection ((**A**): the number of apoptotic cells in each quadrant; (**B**): total apoptosis rate). Solvent control group: 0.3% Dimethylsulfoxide (DMSO); cell model group: cells were incubated with 1 µM Aβ_1–42_ for 24 h; low/medium/high-dose drug protection group: cells were pre-treated with 20/40/60 µg/mL Cyanidin-3-*O*-glucoside for 24 h, followed by incubation with 1 µM Aβ_1–42_ for 24 h, and Cyanidin-3-*O*-glucoside was left during Aβ_1–42_ incubation; drug treatment group: cells were incubated with 40 µg/mL Cyanidin-3-*O*-glucoside and 1 µM Aβ_1–42_ for 24 h, and Cyanidin-3-*O*-glucoside was left during Aβ_1–42_ incubation. Different lowercase letters indicate significant differences (*p* < 0.05).

Figure 8A shows the early and late apoptosis of cells in different groups. Figure 8B shows the total apoptotic rate. As shown in Figure 8B, compared with the control group, the model group showed a significantly higher apoptotic rate (24.25%, *p* < 0.05). Compared with the model group, the drug protection groups showed a significant dose-dependent decrease in the apoptotic rate (*p* < 0.05). The high-dose drug protection group had a significantly higher apoptotic rate (8.03%) than the control group (*p* < 0.05). In addition, the drug treatment group showed significantly lower apoptosis rates (17.62%) than the model group and the medium-dose drug protection group (both *p* < 0.05), but not the high-dose drug protection group (*p* > 0.05). These findings suggest that Cyanidin-3-*O*-glucoside can mitigate the Aβ_1–42_ induced apoptosis in SH-SY5Y cells, with no difference in the protective effect compared with its therapeutic effect.

### 3.7. Western Blot

**Figure 9:**
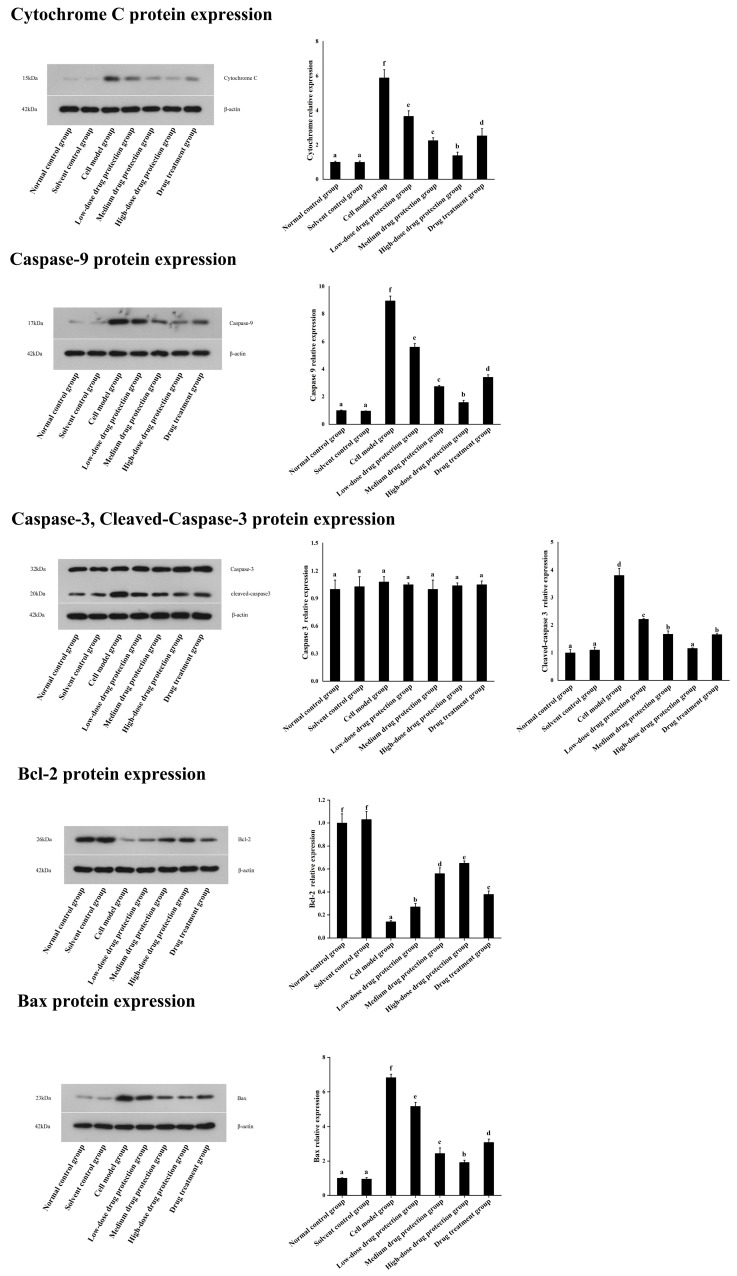
Western blot analysis. Solvent control group: 0.3% Dimethylsulfoxide (DMSO); cell model group: cells were incubated with 1 µM Aβ_1–42_ for 24 h; low/medium/high-dose drug protection group: cells were pre-treated with 20/40/60 µg/mL Cyanidin-3-*O*-glucoside for 24 h, followed by incubation with 1 µM Aβ_1–42_ for 24 h, and Cyanidin-3-*O*-glucoside was left during Aβ_1–42_ incubation; drug treatment group: cells were incubated with 40 µg/mL Cyanidin-3-*O*-glucoside and 1 µM Aβ_1–42_ for 24 h, and Cyanidin-3-*O*-glucoside was left during Aβ_1–42_ incubation. Different lowercase letters indicate significant differences (*p* < 0.05).

As shown in Figure 9, compared with the control group, the model group showed significantly lower expression levels of Bcl-2 protein. Further, compared with the model group, the drug protection groups showed a dose-dependent increase in Bcl-2 protein expression. Moreover, compared with the model group, the treatment group showed upregulated Bcl-2 protein expression. Similarly, compared with the control group, the drug protection groups showed downregulated expression of cytochrome C, caspase 9, cleaved caspase 3, and Bax. These expression levels showed a dose-dependent decrease in the drug protection groups.

### 3.8. RT-PCR

**Figure 10:**
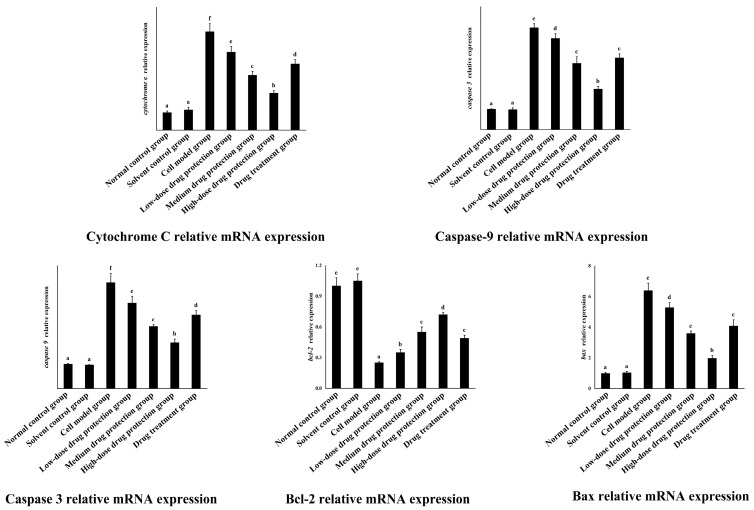
Quantitative Real-time PCR. Solvent control group: 0.3% Dimethylsulfoxide (DMSO); cell model group: cells were incubated with 1 µM Aβ_1–42_ for 24 h; low/medium/high-dose drug protection group: cells were pre-treated with 20/40/60 µg/mL Cyanidin-3-*O*-glucoside for 24 h, followed by incubation with 1 µM Aβ_1–42_ for 24 h, and Cyanidin-3-*O*-glucoside was left during Aβ_1–42_ incubation; drug treatment group: cells were incubated with 40 µg/mL Cyanidin-3-*O*-glucoside and 1 µM Aβ_1–42_ for 24 h, and Cyanidin-3-*O*-glucoside was left during Aβ_1–42_ incubation. Different lowercase letters indicate significant differences (*p* < 0.05).

As shown in Figure 10, the relative mRNA expression level of cytochrome C in the model group was significantly higher than that in the control group (*p* < 0.05). Compared with the model group, the drug protection groups showed a significant dose-dependent decrease in the relative mRNA expression level of cytochrome C and Caspase 3 (*p* < 0.05). The relative mRNA expression of cytochrome C in the drug treatment group was significantly lower than that in the model group and higher than that in the medium-dose drug protection group (*p* < 0.05). The relative mRNA expression level of caspase 3 in the model group was significantly higher than that in the control group (*p* < 0.05). The drug treatment groups showed significantly lower relative mRNA expression levels of caspase 3 compared with the model group (*p* < 0.05) but not the medium-dose drug protection group (*p* > 0.05). The relative mRNA expression level of caspase 9 in the model group was significantly higher than that in the control group (*p* < 0.05). Compared with the model group, the drug protection groups showed a significant dose-dependent decrease in the relative mRNA expression level of caspase 9 (*p* < 0.05). The drug treatment group showed significantly lower relative mRNA expression levels of caspase 9 than the model group and higher expression levels than the medium-dose drug protection group (*p* < 0.05). Compared with the control group, the model group showed significantly lower relative expression levels of Bcl-2 mRNA (*p* < 0.05). Compared with the model group, the drug protection groups showed a significant dose-dependent increase in the relative expression level of Bcl-2 mRNA (*p* < 0.05). The drug treatment group showed a significantly higher relative expression level of Bcl-2 mRNA than the model group (*p* < 0.05) but not the medium-dose drug protection group (E) (*p* > 0.05). Compared with the control group, the model group showed significantly increased mRNA relative expression of Bax (*p* < 0.05). Compared with the model group, the drug protection groups showed a dose-dependent decrease in the relative expression level of Bax mRNA. The drug treatment group showed significantly lower relative expression of Bax mRNA than the model group (*p* < 0.05) but not the medium-dose drug protection group (*p* > 0.05).

## 4. Discussion

Oxidative stress and apoptosis are crucially involved in AD pathogenesis, and oxidative stress can induce apoptosis [20,21]. Additionally, mitochondrial dysfunction related to Ca^2+^ homeostasis is crucially involved in apoptosis [22]. Increased intracellular Ca^2+^ levels are considered a key indicator of apoptosis that regulates multiple Ca^2+^-dependent proteases and activates mitochondrial pathways [23,24]. Reports on the effects of anthocyanins on AD have focused on mixtures. Low-purity anthocyanins cannot allow elucidation of the mechanisms underlying the intervention effect of anthocyanins on AD [19,25]. Studies on the intervention effect of the Cyanidin 3-*O*-glucoside monomer on AD have focused on oxidative stress, apoptosis, and inflammation. Meng et al. [17] examined the preventive effect of Cyanidin 3-*O*-glucoside on SH-SY5Y cells through the Nrf2 pathway. Studies have examined the relationship between inflammation and AD [26]; furthermore, Pereira et al. [27] assessed the anti-inflammatory effects of the Cyanidin-3-*O*-glucoside monomer. Zhang et al. [28] confirmed through in vitro and in vivo experiments that Cyanidin-3-*O*-glucoside can alleviate Aβ25-35-induced damage of SH-SY5Y cells through the PPARγ pathway and increase the cognitive ability of APPswe/PSEN1dE9 double transgenic AD mice in a novel object recognition experiment. You et al. reported that Cyanidin-3-*O*-glucoside could restore reactive oxygen species (ROS) and mitochondrial membrane potential levels in PC12 cells altered by Aβ 25–35 treatment. JNK and P38 phosphorylation are inhibited by stimulating the release of cytochrome C and AIF into the cytoplasm as well as reducing the activation of Caspase-3, Caspase-8, and Caspase-9, which inhibits apoptosis [29]. Cyanidin-3-*O*-glucoside has a very good effect on AD. However, the prevention and treatment of AD by regulating the calcium-mediated mitochondrial apoptosis pathway remains unclear.

In our study, the model group showed significantly higher intracellular calcium levels than the control, drug protection, and treatment groups. As mentioned previously, the increase in Ca^2+^ levels may cause mitochondrial dysfunction; therefore, we examined ROS, ATP, and mitochondrial membrane potentials. Compared with the control group, the model group showed significantly increased ROS levels as well as significantly decreased ATP levels and mitochondrial membrane potentials. Compared with the model group, the drug protection and treatment groups showed significantly decreased ROS levels as well as significantly increased ATP levels and mitochondrial membrane potentials. Additionally, since mitochondrial dysfunction can cause apoptosis, we analyzed apoptosis itself. We found that Cyanidin-3-*O*-glucoside exerted a dose-dependent preventive effect on Aβ_1–42_-induced apoptosis of SH-SY5Y cells. The protective effects of Cyanidin-3-*O*-glucoside were further explored. Compared with the control group, the model group showed increased expression of cytochrome C, caspase 9, caspase 3, Bax, and other proteins and mRNA as well as decreased Bcl-2 protein and mRNA expression. Compared with the model group, the drug protection groups showed decreased expression levels of cytochrome C, caspase 9, caspase 3, Bax, and other proteins and mRNA as well as increased expression of Bcl-2 protein and mRNA. In the drug protection group, as the dose of Cyanidin-3-*O*-glucoside increased, the apoptosis rate decreased. However, at the same dose of Cyanidin-3-*O*-glucoside, there was no significant change in the apoptosis rate between the drug protection group and the treatment group. Furthermore, Aβ has been shown to form a channel with permeability to calcium ions that is embedded in the lipid bilayer membrane structure. Given the low cytoplasmic calcium levels, even a very low concentration of calcium ions increases the cytoplasmic calcium levels, which disturbs the normal calcium homeostasis in the cell. As an important second messenger, increased calcium ion levels can cause neuronal hyperexcitability leading to neuronal apoptosis and necrosis [30]. Additionally, calcium channel formation is long-lasting [31]. In this process, given the compensatory effect of the brain, it often does not present corresponding symptoms. Considerable ion channel accumulation disturbs the plasma membrane integrity and disrupts calcium homeostasis. At this time, although corresponding symptoms occur, channel generation is irreversible. Hence, Cyanidin-3-*O*-glucoside has a better preventive than therapeutic effect.

Cytochrome c is a basic component in the respiratory chain and is crucially involved in redox and energy metabolism. Additionally, cytochrome c is an essential component of mitochondria and plays a key role in initiating the apoptosis program and in amplifying the apoptotic signal [32]. The two main pathways of apoptosis are the death receptor and mitochondrial apoptosis pathway; moreover, mitochondrial dysfunction related to the loss of Ca^2+^ homeostasis and the resulting activation of caspases play a major role in cell apoptosis [33,34,35]. The site of action of Bcl-2 family proteins is in the outer mitochondrial membrane; further, Bcl-2 protein expression directly determines the permeability of the mitochondrial membrane structure and apoptosis [36]. In normal cells, pro-apoptotic and anti-apoptotic proteins in the Bcl-2 family exist in the form of a heterodimer. However, when normal cells are stimulated, Bax, which is a member of the Bcl-2 protein family, dephosphorylates and dissociates from its inactive complex formed by binding with molecular chaperone proteins, destroying the heterodimer structure. As a result, numerous pro-apoptotic proteins (Bcl-2-associated X [Bax]) accumulate on the outer mitochondrial membrane, which causes excessive opening of mitochondrial membrane permeability transition pores, releasing cytochrome c into the cytoplasm, which activates the caspase cascade reaction to subsequently induce apoptosis [37]. Bax overexpression can inhibit the protective effect of Bcl-2. In addition, based on the RT-PCR detection results, we found that Cyanidin-3-*O*-glucoside can downregulate the expression levels of genes such as cytochrome C, caspase 9, caspase 3, and Bax and the upregulation of Bcl-2 gene expression. Taken together, Cyanidin-3-*O*-glucoside can maintain Ca^2+^ homeostasis, regulate mitochondrial dysfunction and the expression of Bax and the Bcl-2 gene, and prevent the caspase cascade reaction caused by the release of cytochrome c. Accordingly, Cyanidin-3-*O*-glucoside exerts preventive and protective effects against AD.

## 5. Conclusions

In conclusion, we found that Cyanidin-3-*O*-glucoside could mitigate Aβ_1–42_-induced apoptosis of SH-SY5Y cells. However, the therapeutic effect was not as good as the prevention effect. Our findings demonstrated the mechanism underlying the prevention and treatment of Aβ_1–42_-induced apoptosis in SH-SY5Y cells. Further, our findings could inform the potential application of Cyanidin-3-*O*-glucoside in the prevention and treatment of AD and provide a new treatment strategy for AD as well as a theoretical basis for the future development of novel drugs. In addition, Guizhou contains abundant blueberry resources, and functional substances such as anthocyanins are worth further exploration and research.

## Figures and Tables

**Table 1:** Main reagents and manufacturing companies.

Name of the Reagent	Article Number	Production Company	Place of Origin
Aβ_1–42_	/	Gill Biochemical Co., Ltd.	Shenyang, China
MTT	WLA021a	All creatures	Shenyang, China
Reactive oxygen species detection kit	WLA131	All creatures	Shenyang, China
MitoSOX Red Mitochondrial Superoxide Indicator	M36008	Lloyds Kang biological	Shenyang, China
Mitochondrial membrane potential detection kit	C2006	Blue skies	Shenyang, China
ATP detection KitBCA protein concentration detection kit	S0026	Blue skies	Shenyang, China
Calcium checkerboard	WLA004a	Wanleibio	Shenyang, China
Apoptosis detection kit TRIpureBeyoRT I1M-MLV reverse transcription	RP1001	BioTeke	Beijing, China
enzyme	D7160L	Blue skies	Shanghai, China
RNase inhibitor	RP5602	BioTeke	Beijing, China
2 × Taq PCR MasterMix	PC1150	Solarbio	Beijing, China
SYBR Green	SY1020	Solarbio	Beijing, China
Cytochrome C antibody	WL02410	Wanleibio	Shenyang, China
Bcl-2 antibody	WL01556	Wanleibio	Shenyang, China
Bax antibody	WL01637	Wanleibio	Shenyang, China
Caspase-9 antibody	WL01838	Wanleibio	Shenyang, China
Caspase3/cleaved-Caspase3 antibody	WL02117	Wanleibio	Shenyang, China
Sheep Anti-Rabbit LG-HRP Internal	WLA023	Wanleibio	Shenyang, China
Reference antibody P-actin total	WL01372	Wanleibio	Shenyang, China
	WLA019	Wanleibio	Shenyang, China
Protein extraction kit MEM medium	41500	Mr. Lai treasure	Shenyang, China
F12 medium	BL311A	Biosharp	Shenyang, China
Fetal bovine serum	11011-8611	Sijiqing	Shenyang, China
PBS	B548117	Sangon	Shenyang, China
Pancreatic enzyme	T4799	Sigma	Shenyang, China
EDTA	E6758	Sigma	Shenyang, China
Sodium Pyruvate	S104174	Aladdin	Shenyang, China
Gluta-max	35050079	Syme Fly	Shenyang, China
Rhod-2 AM probe	MX4507	Shanghai Maokang Biological	Shenyang, China
Pluronic F127	P6790	Mr. Lai treasure	Shenyang, China
MitoTracker Green probe	C1048	Blue skies	Shenyang, China

HRP, horse radish peroxidase; MEM, minimum essential medium; PBS, phosphate-buffered saline; EDTA, ethylene-diamine-tetra-acetic acid.

**Table 2:** Main instrument and manufacturing company.

Instrument Used	Manufacturing Company	Place of Origin	Company
Ultra-pure water system	NW10LVF	Hong Kong	Heal Force
Ultra-high speed refrigerated centrifuge	H-2050R	Changsha, China	Hunan instrument
CO_2_ incubator inverted	HF-90	Shanghai, China	Shanghai force
Phase contrast microscope ultra clean table	IX53	Shanghai, China	OLYMPUS
Enzyme standard instrument	800TS	Shanghai, China	BIOTEK
Flow cytometer	NovoCyte	Shanghai, China	Agilent
Multifunctional enzyme marker	SynergyH1	Shanghai, China	Biotek
Micropipette	Proline	Suzhou, China	BIOHIT
Ultraviolet visible spectrophotometer	UV752N	Shanghai, China	Shanghai saso
Digital display constant temperature water bath	HH-4	Jintan, China	The splendor instrument
Vacuum drying oven	DZF-6050	Shanghai, China	SYSBERY
Fluorescence quantitative PCR instrument	Exicycler 96	Shanghai, China	BIONEER
Electrophoresis apparatus	DYY-7C	Beijing, China	Beijing, June 1
Transfer trough	DYCZ-40D	Beijing, China	Beijing, June 1
Double vertical protein Electrophoresis gel	DYCZ-24DN	Beijing, China	Beijing, June 1
Imaging system	WD-9413B	Beijing, China	Beijing, June 1
Overspeed refrigerated Centrifuge electric	H-2050R	Changsha, China	Hunan instrument
Thermostatic incubator	DH36001B	Tianjin, China	Tianjin Tester
Calcium testing box	C004	Nanjing, China	Nanjing Jiancheng Co., Ltd.

## Data Availability

The datasets used and analyzed during the current study are available from the corresponding author on reasonable request.
